# Antibiotic-Resistant Enterococci and Fecal Indicators in Surface Water and Groundwater Impacted by a Concentrated Swine Feeding Operation

**DOI:** 10.1289/ehp.9770

**Published:** 2007-03-22

**Authors:** Amy R. Sapkota, Frank C. Curriero, Kristen E. Gibson, Kellogg J. Schwab

**Affiliations:** 1 Department of Environmental Health Sciences, Johns Hopkins Bloomberg School of Public Health, Baltimore, Maryland, USA; 2 Maryland Institute for Applied Environmental Health, College of Health and Human Performance, University of Maryland, College Park, Maryland, USA; 3 Department of Biostatistics, Johns Hopkins Bloomberg School of Public Health, Baltimore, Maryland, USA

**Keywords:** antibiotic resistance, CAFO, concentrated swine feeding operation, *E. coli*, enterococci, fecal coliforms, fecal indicators, groundwater, surface water

## Abstract

**Background:**

The nontherapeutic use of antibiotics in swine feed can select for antibiotic resistance in swine enteric bacteria. Leaking swine waste storage pits and the land-application of swine manure can result in the dispersion of resistant bacteria to water sources. However, there are few data comparing levels of resistant bacteria in swine manure–impacted water sources versus unaffected sources.

**Objectives:**

The goal of this study was to analyze surface water and groundwater situated up and down gradient from a swine facility for antibiotic-resistant enterococci and other fecal indicators.

**Methods:**

Surface water and groundwater samples (*n* = 28) were collected up and down gradient from a swine facility from 2002 to 2004. Fecal indicators were isolated by membrane filtration, and enterococci (*n* = 200) were tested for susceptibility to erythromycin, tetracycline, clindamycin, virginiamycin, and vancomycin.

**Results:**

Median concentrations of enterococci, fecal coliforms, and *Escherichia coli* were 4- to 33-fold higher in down-gradient versus up-gradient surface water and groundwater. We observed higher minimal inhibitory concentrations for four antibiotics in enterococci isolated from down-gradient versus up-gradient surface water and groundwater. Elevated percentages of erythromycin- (*p* = 0.02) and tetracycline-resistant (*p* = 0.06) enterococci were detected in down-gradient surface waters, and higher percentages of tetracycline- (*p* = 0.07) and clindamycin-resistant (*p* < 0.001) enterococci were detected in down-gradient groundwater.

**Conclusions:**

We detected elevated levels of fecal indicators and antibiotic-resistant enterococci in water sources situated down gradient from a swine facility compared with up-gradient sources. These findings provide additional evidence that water contaminated with swine manure could contribute to the spread of antibiotic resistance.

In 2005, the United States produced > 103 million pigs at 67,000 production facilities [U.S. Department of Agriculture (USDA) 2006a, 2006b]. Facilities housing > 55,000 pigs accounted for more than half of the total U.S. swine inventory, reflecting the increasing consolidation and concentration of U.S. swine production (USDA 2006a). This trend in swine production has resulted in the concentration of large volumes of manure in relatively small geographic areas. Manure is typically stored in deep pits or outdoor lagoons and then applied to agricultural fields as a source of fertilizer. However, as a result of runoff and percolation events, components of manure, including human pathogens and chemical contaminants, can affect surface water and groundwater proximal to swine concentrated animal feeding operations (CAFOs), posing risks to human health (Anderson and Sobsey 2006; Campagnolo et al. 2002; Jongbloed and Lenis 1998; Krapac et al. 2002; Sayah et al. 2005; Thurston-Enriquez et al. 2005). Specific swine production practices, including the use of nontherapeutic levels of antibiotics in swine feed, can exacerbate the risks associated with exposures to manure-contaminated water sources.

An estimated 10.3 million pounds of antibiotics are used annually in U.S. swine production for nontherapeutic purposes such as promoting growth and improving feed efficiency (Mellon et al. 2001). These antibiotics are the same drugs that are used in human clinical medicine and include tetracycline, erythromycin, lincomycin, virginiamycin, and ampicillin, to name a few [U.S. Food and Drug Administration (FDA) 2004]. The practice of administering nontherapeutic levels of antibiotics in swine feed selects for antibiotic resistance among commensal and pathogenic bacteria in swine (Aarestrup et al. 2000; Bager et al. 1997; Wegener 2003), resulting in high levels of resistant bacteria and resistance genes in swine waste (Chee-Sanford et al. 2001; Haack and Andrews 2000; Parveen et al. 2006). Haack and Andrews (2000) detected 1.6 × 10^7^ colony forming units (CFU)/mL of total tetracycline-resistant bacteria and 2.1 × 10^5^ CFU/mL of tetracycline-resistant enterococci in swine waste. Parveen et al. (2006) identified resistance to at least one antibiotic in 85% of *Escherichia coli* isolates recovered from a swine lagoon. In addition, Chee-Sanford et al. (2001) detected up to eight known tetracycline resistance genes in total DNA extracted from swine lagoon samples. In the same study, a broad range of tetracycline resistance determinants were found in groundwater samples collected downstream of swine lagoons (Chee-Sanford et al. 2001). Anderson and Sobsey (2006) also detected higher percentages of antibiotic-resistant *E. coli* in groundwater collected in the vicinity of large-scale swine facilities compared with groundwater collected at reference sites. In another study, Sayah et al. (2005) found that 80.6% of *E. coli* isolates collected from surface waters located near swine and other livestock facilities were resistant to at least one antibiotic.

The presence of swine-associated resistant bacteria in rural surface water and ground-water sources is important to human health because exposure to these sources could enable the transfer of resistant bacteria from swine to humans, contributing to the spread and persistence of antibiotic resistance. However, beyond the studies of Chee-Sanford et al. (2001), Anderson and Sobsey (2006), and Sayah et al. (2005), there are few data in the published literature regarding the presence of antibiotic-resistant bacteria in surface waters and groundwater located in the vicinity of swine CAFOs. Moreover, there are few data available comparing concentrations of fecal indicators in groundwater and surface waters impacted by swine CAFOs compared with unaffected waters. Thus, the goal of this study was to analyze surface water and groundwater samples collected up gradient and down gradient from a swine CAFO for the presence of antibiotic-resistant enterococci. Enterococci are commensal bacteria (as well as opportunistic pathogens) that are found in the intestinal tracts of animals and humans and are often used as indicators of fecal contamination in water sources [U.S. Environmental Protection Agency (U.S. EPA) 2000]. The presence of other fecal indicators, including fecal coliforms and *E. coli,* was also investigated in surface water and groundwater samples collected throughout this study.

## Materials and Methods

### Study site

**Figure 1:**
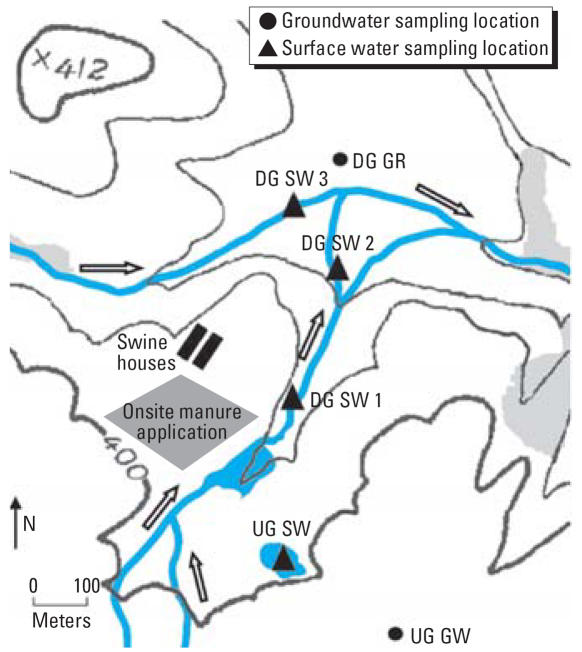
Map of study site and sampling locations. Abbreviations: DG GR, down-gradient groundwater sampling location; DG SW 1, first down-gradient surface water sampling location; DG SW 2, second down-gradient surface water sampling location; DG SW 3, third down-gradient surface water sampling location; UG GW, up-gradient groundwater sampling location; UG SW, up-gradient surface water sampling location. Topographic contour lines are given in feet, and contour intervals = 20 vertical ft. Arrows indicate the direction of surface water flow. Topographic data were obtained from a U.S. Geological Survey map of the study area (U.S. Geological Survey 2006).

This study was conducted around a swine finishing CAFO located in a rural area in the Mid-Atlantic United States (Figure 1). The CAFO is composed of two tunnel-ventilated swine houses, and the full day-to-day capacity of the entire facility is 5,000 hogs. However, throughout the sampling period, approximately 3,000 hogs were present at the facility. Manure wastes from the CAFO are stored in 12-ft deep concrete manure pits that lie beneath each swine house. Once the pits are filled to maximum capacity, the waste is siphoned off and applied to agricultural fields both on-site (Figure 1) and off-site. At this facility, nontherapeutic levels of antibiotics are administered in swine feed; however, specific usage data could not be obtained from the swine grower.

### Sample collection

Surface water and groundwater samples were collected during six sampling trips that took place between 2002 and 2004 (Table 1). A total of 15 surface water samples were collected from three locations situated down gradient from the swine CAFO, and a total of 4 surface water samples were recovered from one location situated up gradient from the swine CAFO (Figure 1). As indicated in Figure 1, the down-gradient surface water sampling locations were situated in a stream system that was likely affected by surface water runoff events from the swine CAFO. Sampling locations on two different, connecting tributaries in this stream system were chosen in order to determine the impacts of the swine CAFO on both of these tributaries. Down-gradient surface water samples were collected only when there was adequate flow at a sampling location such that water samples could be collected into 1-L sampling bottles in an upstream motion, midway between the surface and the stream bottom, without disturbing bottom sediment. We were unable to obtain access to an up-gradient surface water sampling location situated within the same stream system because *a*) we could not penetrate dense and deep thickets that completely surrounded the stream (on accessible property) without making major modifications to existing vegetation; or *b*) we were not allowed access to personal property farther upstream. Because of these challenges, we identified an up-gradient pond located on accessible property (Figure 1) to serve as an up-gradient surface water control site that was not affected by the swine CAFO.

Groundwater samples were collected from one drinking water well situated down gradient from the swine CAFO (*n* = 4) and one drinking water well situated up gradient from the swine CAFO (*n* = 5) (Figure 1). Both wells are located in the Piedmont Plateau Province of the Mid-Atlantic United States in an area characterized by unmetamorphosed bedrock composed of red shale. The up-gradient well was constructed in 1990 and is used as a primary source of drinking water by the property owners. It is 250 ft deep and lined with steel casing to a depth of 56 ft. Water is encountered at depths of 185 ft and 228 ft. The down-gradient well is an older well that was used as a primary source of drinking water by the property owners before the neighboring swine CAFO was built. Information on the precise depth and construction of this well was unavailable; however, groundwater on the property is encountered at depths of approximately 90 ft and 132 ft. None of the wells were subject to any disinfection before sampling; at each well, water was flushed for 1 min before groundwater samples were collected.

A manure pit sample was collected directly from the manure pits during one sampling trip in January 2004. All surface water, groundwater, and manure pit samples were collected in 1-L sterile Nalgene Wide Mouth Environmental Sample Bottles (Nalgene, Lima, OH); labeled; and transported back to the laboratory at 4°C. Sample processing took place within 3–6 hr after sample collection.

### Isolation and enumeration of fecal indicators

*Enterococcus* spp., *E. coli*, and fecal coliforms were isolated from each water sample using standard membrane filtration methods: U.S. Environmental Protection Agency (EPA) Method 1106.1 and Method 1103 (U.S. EPA 2000), and standard method SM 9222D [American Public Health Association (APHA) 1998]. Briefly, 10-fold dilutions of each water sample were prepared (10^0^, 10^−1^, 10^−2^, and 10^−3^), and 10 mL of each dilution were filtered through 0.45-μm, 47-mm mixed cellulose ester filters (Millipore, Billerica, MA), which were placed onto appropriate agar plates. We used mE agar for the detection and enumeration of *Enterococcus* spp., mTEC agar for the detection of *E. coli*, and mFC agar for the detection of fecal coliforms (all from Becton Dickinson, Sparks, MD). Negative control filters and negative control agar plates were included in each membrane filtration analysis. Incubation conditions for the agar plates were as follows: mE plates, 41.5°C for 48 hr; mTEC plates, 35°C for 2 hr followed by 44.5°C for 22 hr; and mFC plates, 44.5°C for 24 hr. After 24 hr, membrane filters from mTEC agar plates were placed in 1.2 mL urea for 5 min; bright yellow colonies were considered presumptive *E. coli*. Blue colonies arising on the mFC agar plates were considered presumptive fecal coliforms. After 48 hr, membrane filters from mE agar plates were placed on esculin iron agar (EIA) plates and incubated at 41.5°C for 20 min. Colonies characteristic of *Enterococcus* spp., ranging from pink to dark red on mE agar and producing a brown to black precipitate on EIA agar, were considered presumptive *Enterococcus* spp. (U.S. EPA 2000). All resulting colonies were counted, and concentrations of *Enterococcus* spp., *E. coli,* and fecal coliforms per 100 mL water were determined from dilution plates containing 30–300 CFU using back calculations. One to 10 presumptive *Enterococcus* spp. recovered from each sample were archived in tryptic soy broth with 20% glycerol at −80°C for additional analyses.

### *Identification of *Enterococcus *spp*

Presumptive *Enterococcus* spp. (*n* = 200) were identified to the species level using isolation and identification procedures described previously (Chapin et al. 2005; Murray et al. 2003). *Enterococcus faecalis* 29212 and *Enterococcus faecium* 19434 (American Type Culture Collection, Manassas, VA) were included as quality control strains. Briefly, all isolates and control strains were streaked from −80°C archived stocks onto tryptic soy agar No. 2 amended with 5% defibrinated sheep blood (Quad Five, Ryegate, MT), and incubated for 24 hr at 37°C. Gram-positive cocci were verified by Gram stains, and the production of catalase was tested for each isolate in the presence of 3% hydrogen peroxide. All isolates were negative for catalase activity and were further tested for pyrolidonyl-arylamidase (PYRase) activity using Remel’s PYR kit (Remel, Lenexa, KY). All isolates were also PYRase-positive and were distinguished further by testing for the reduction of tellurite. Isolates and quality control strains were streaked from −80°C archived stocks onto nutrient agar with 0.4% potassium tellurite (Sigma-Aldrich Corp., St. Louis, MO) and incubated for 24–72 hr at 37°C. Isolates producing a black precipitate were considered positive for tellurite reduction and identified as *E. faecalis*. The remaining isolates were identified using the following standard biochemical tests: lactose, sucrose, arabinose, sorbitol, raffinose, and mannitol carbohydrate fermentation; deamination of arginine; methyl-α-D-glucopyranoside acidification; utilization of pyruvate; and pigmentation of the isolate.

### Antimicrobial susceptibility testing

We used the minimal inhibitory concentration (MIC) agar dilution method [Clinical and Laboratory Standards Institute (CLSI) 2002] to test antimicrobial susceptibility among the *Enterococcus* spp. (*n* = 200). *E. faecalis* 29212 was included as the quality control reference strain. We tested susceptibility to the following antibiotics: erythromycin, clindamycin, tetracycline, and virginiamycin (streptogramin A and B combination), all of which are approved for use in U.S. swine production (FDA 2004); and vancomycin, which has never been approved for use in U.S. livestock. All antibiotics were obtained from Sigma-Aldrich (St. Louis, MO), except for virginiamycin, which was purchased from Research Products International Corp. (Mt. Prospect, IL). The following concentrations of antibiotics were tested: 0.5–256 μg/mL erythromycin, 0.5–256 μg/mL tetracycline, 0.03–256 μg/mL clindamycin, 0.03–64 μg/mL virginiamycin, and 0.03–64 μg/mL vancomycin. These antibiotic test ranges were chosen to include the MIC quality control ranges of the reference strain (*E. faecalis* 29212), the antibiotic resistance break points established by the CLSI for enterococci (CLSI 2002), and antibiotic concentrations that exceeded resistance break points by at least 2-fold.

In preparation for the MIC agar dilution tests, all *Enterococcus* spp. isolates were streaked onto plates containing tryptic soy agar No. 2 amended with 5% defibrinated sheep blood (QuadFive, Rygate, MT), and incubated at 37°C for 24 hr. Each isolate was then suspended in 3 mL Mueller-Hinton broth and adjusted to a 0.5 McFarland standard using a Vitek colorimeter (Hach, Loveland, CO). Two hundred microliters of each suspension were loaded into individual wells within a Cathra replicator plate (Oxoid Inc., Ogdensburg, NY) and replicated onto a series of Mueller-Hinton agar plates amended with 2-fold increasing antibiotic concentrations. Plates were incubated at 37°C for 24 hr, and MICs were subsequently recorded as the minimum concentration of antibiotic that completely inhibited growth. Each isolate was categorized using the following MIC resistance breakpoints established for *Enterococcus* spp. by the CLSI (2002): erythromycin, ≥ 8 μg/mL; clindamycin, ≥ 4 μg/mL; tetracycline, ≥ 16 μg/mL; virginiamycin, ≥ 4 μg/mL; and vancomycin ≥ 32 μg/mL.

### Statistical analyses

We compared concentrations of fecal indicators (*Enterococcus* spp., *E. coli,* and fecal coliforms) between up-gradient and down-gradient surface water samples and up-gradient and down-gradient groundwater samples using two-sample Wilcoxon rank-sum tests. Fisher’s exact tests were used to compare rates of erythromycin-, tetracycline-, clindamycin-, virginiamycin-, and vancomycin-resistant *Enterococcus* spp. between up-gradient and down-gradient surface water samples and up-gradient and down-gradient groundwater samples. For the surface water analyses, data obtained from the three surface water sampling locations situated down gradient from the swine CAFO were pooled because these sites did not represent a significant source of variation in the data. Specifically, levels of fecal indicators, patterns of antimicrobial resistance, and geographic proximity were comparable among all samples obtained from these locations (data not shown), providing evidence for a shared source. Since *E. faecalis* can be intrinsically resistant to clindamycin and virginiamycin (Singh and Murray 2005), analyses comparing rates of clindamycin resistance and virginiamycin resistance were restricted to non–*E. faecalis* isolates. All statistical analyses were performed using Intercooled Stata 7.0 (Stata Corporation, College Station, TX).

## Results

### Concentrations of fecal indicators

Median concentrations of *Enterococcus* spp., *E. coli,* and fecal coliforms were 17-, 11- and 33-fold higher, respectively, in surface waters located down gradient of the swine CAFO compared with surface waters located up gradient of the CAFO; the differences were statistically significant (*p* = 0.003, 0.007, and 0.010, respectively) (Table 2). Likewise, median concentrations of *Enterococcus* spp., *E. coli,* and fecal coliforms were 4-, 11-, and 20-fold higher, respectively, in down-gradient groundwater samples versus up-gradient groundwater samples (*p* = 0.085, 0.007, and 0.007, respectively) (Table 2). Concentrations of *Enterococcus* spp., *E. coli,* and fecal coliforms found in manure pit samples were 5.2 × 10^5^ CFUs/100 mL, 1.0 × 10^6^ CFUs/100 mL, and 8.8 × 10^6^ CFUs/100 mL, respectively.

### Enterococcus spp. isolated from water and manure pit samples

A variety of *Enterococcus* spp. was identified in groundwater, surface water, and manure pit samples (Table 3). *E. faecalis* was the predominant species isolated from all sample types, representing 67% of all *Enterococcus* spp. that were analyzed for antibiotic susceptibility in this study. For 29 (14.5%) of the *Enterococcus* spp., results from the standard biochemical identification tests were not completely consistent with known species of enterococci. These isolates could only be identified to the genus level and are listed as “other *Enterococcus* spp.” in Table 3.

### Antibiotic resistance

Overall, higher erythromycin and tetracycline MICs were detected among *Enterococcus* spp. (*E. faecalis* and non–*E. faecalis*) recovered from down-gradient groundwater and surface water samples compared with up-gradient groundwater and surface water samples (Table 4). For example, erythromycin MIC_90s_ (MIC required to inhibit the growth of 90% of organisms) for *Enterococcus* spp. recovered from down-gradient groundwater and surface water samples were at least 4-fold and 128-fold higher, respectively, than that of isolates recovered from up-gradient groundwater and surface water samples. These data suggest that down-gradient surface water and groundwater sources are contaminated with *Enterococcus* spp. that express higher levels of erythromycin and tetracycline resistance. The highest erythromycin and tetracycline MICs were observed among *Enterococcus* spp. recovered from manure pits, where erythromycin and tetracycline MIC_90s_ were > 256 μg/mL and 179.2 μg/mL, respectively (Table 4). In contrast, MICs for vancomycin, a drug that has never been approved for use in U.S. swine production, were generally below the CLSI vancomycin resistance breakpoint of ≥ 32 μg/mL (CLSI 2002) among *Enterococcus* spp. recovered from all sample types. The exceptions were isolates recovered from up-gradient groundwater samples, which exhibited elevated vancomycin MICs (Table 4).

Similar to the findings for erythromycin and tetracycline, higher clindamycin and virginiamycin MICs were observed among non–*E. faecalis* isolates recovered from down-gradient groundwater and surface water samples compared with up-gradient groundwater and surface water samples (Table 5). For instance, clindamycin MIC_90s_ for non–*E. faecalis* isolated from down-gradient groundwater and surface water samples were at least 2,133-fold and 2-fold higher, respectively, than that of non–*E. faecalis* recovered from up-gradient groundwater and surface water samples. The highest clindamycin and virginiamycin MICs were observed among isolates recovered from manure pits (Table 5). As anticipated, clindamycin and virginiamycin MICs among *E. faecalis*—which have been shown to be intrinsically resistant to both of these antibiotics (Singh and Murray 2005)—were similar among isolates recovered from all sample types, except in the case of *E. faecalis* recovered from manure pits. These isolates exhibited higher levels of both clindamycin and virginiamycin resistance (Table 5).

In comparing the percentage of antibiotic-resistant *Enterococcus* spp. present in up-gradient versus down-gradient surface water samples, higher percentages of erythromycin-, tetracycline-, virginiamycin-, and vancomycin-resistant isolates were observed in down-gradient versus up-gradient surface waters (Table 6). In contrast, we observed a higher percentage of clindamycin-resistant isolates in up-gradient surface water samples. However, using Fisher’s exact test, we found that only the elevated percentage of erythromycin-resistant isolates found in down-gradient surface water samples was statistically significant (*p* = 0.02) (Table 6). The higher percentage of tetracycline-resistant isolates observed in down-gradient surface water samples was marginally significant (*p* = 0.06) (Table 6).

In groundwater samples, higher percentages of tetracycline- and clindamycin-resistant *Enterococcus* spp. were observed in down-gradient versus up-gradient groundwater samples (Table 6). The elevated percentage of clindamycin-resistant isolates in down-gradient groundwater samples was highly statistically significant (*p* < 0.001), whereas the higher percentage of tetracycline-resistant isolates in down-gradient groundwater samples was marginally significant (*p* = 0.07) (Table 6). Conversely, higher percentages of erythromycin- and vancomycin-resistant *Enterococcus* spp. were observed in up-gradient versus down-gradient groundwater samples, and the differences in erythromycin resistance were statistically significant (*p* < 0.001).

## Discussion

In this study we investigated surface water and groundwater located up gradient and down gradient of a swine CAFO for the presence of fecal indicators (*Enterococcus* spp., *E. coli,* and fecal coliforms) and antibiotic-resistant enterococci. Findings indicate that surface waters and groundwater located down gradient of the swine CAFO are contaminated with significantly higher levels of *Enterococcus* spp., *E. coli,* and fecal coliforms compared with surface water and groundwater located up gradient of the swine CAFO (Table 2). The groundwater data are in agreement with two previous studies that examined groundwater wells situated near large-scale swine facilities (Anderson and Sobsey 2006; Krapac et al. 2002). Anderson and Sobsey (2006) detected *E. coli* at a range of 0.5–32.7 CFU/100 mL in groundwater samples collected at two large-scale swine facilities in North Carolina. Krapac et al. (2002) detected fecal coliforms at a maximum concentration of 7 CFU/100 mL in shallow ground-water samples collected at a swine finishing facility in Illinois. In addition, Krapac et al. (2002) detected fecal streptococcus in more groundwater samples and at higher concentrations than fecal coliforms. Similarly, we identified *E. coli* and fecal coliforms in down-gradient groundwater samples at ranges of 3–40 CFU/100 mL and 3–70 CFU/100 mL, respectively, and *Enterococcus* spp. (members of the fecal streptococcus group) were consistently detected at higher concentrations than fecal coliforms (Table 2). To our knowledge, the surface water data presented here are the first data to compare levels of fecal indicators in up-gradient versus down-gradient surface waters located in the proximity of a swine CAFO.

The presence of *Enterococcus* spp., *E. coli,* and fecal coliforms in rural surface water and groundwater sources impacted by swine CAFOs may pose health risks to people who either recreate in contaminated surface waters or use the groundwater as a drinking water source. Concentrations of *Enterococcus* spp. and *E. coli* in down-gradient surface water samples collected in this study were consistently in excess of the following U.S. EPA bacterial water quality standards for recreational fresh waters: *Enterococcus* spp., 33 CFU/ 100 mL; and *E. coli*, 126 CFU/100 mL (U.S. EPA 2003). Throughout the sampling period for this study, young children were observed swimming and playing in surface waters located within 500 m down gradient of the swine CAFO; these children could have been exposed to elevated concentrations of *Enterococcus* spp., *E. coli,* and other more harmful microorganisms that may have been present. In addition, if the down-gradient private well tested in this study was part of a public drinking-water-system testing program, it consistently would be in violation of current maximum contaminant level standards for total coliforms (including fecal coliforms and *E. coli*) (U.S. EPA 2002). On each sampling trip, this down-gradient well tested positive for both fecal coliforms and *E. coli.* Before the swine CAFO began production, the owners of this well relied on it as their sole source of drinking water. However, after the facility reached a full working capacity of 5,000 hogs, the owners told us that they had their well tested by an independent, certified laboratory and the water was subsequently deemed nonpotable.

The results of this study also emphasize that human health risks associated with exposures to surface water and groundwater situated down gradient of swine CAFOs could be exacerbated by the presence of antibiotic-resistant bacteria. Overall findings indicate that *Enterococcus* spp. recovered from down-gradient surface water and groundwater samples express higher levels of resistance (higher MICs) to antibiotics that are commonly used in both swine production and human clinical medicine (erythromycin, tetracycline, clindamycin, and virginiamycin) compared with *Enterococcus* spp. recovered from up-gradient surface water and groundwater samples (Tables 4 and 5). In contrast, *Enterococcus* spp. recovered from all sample types (down-gradient water samples, up-gradient water samples, and manure samples) were, in general, similarly susceptible to vancomycin (Table 4), a drug that has never been approved for use in U.S. swine production.

The patterns of antibiotic resistance observed in *Enterococcus* spp. recovered from down-gradient surface water and groundwater samples were similar to those observed in isolates recovered from manure pit samples, particularly resistance patterns associated with erythromycin, tetracycline, and clindamycin (Tables 4 and 5). We also have reported similar patterns of erythromycin, tetracycline, and clindamycin resistance among *Enterococcus* spp. recovered from indoor air samples collected within the same swine CAFO during the same sampling period (Chapin et al. 2005). These data support previous findings of Chee-Sanford et al. (2001) showing that the movement of resistant bacteria and resistance determinants from swine CAFOs into the environment can be extensive. Chee-Sanford et al. (2001) found a high occurrence of tetracycline resistance determinants in groundwater wells located close to swine lagoons; however, they also detected one resistance determinant in a well situated over 250 m downstream of one of the lagoons. In the present study, antibiotic-resistant *Enterococcus* spp. were detected in a drinking water well located 400 m down gradient of a swine CAFO, as well as in surface water situated 300 m down gradient from the facility (Figure 1). The presence of resistant bacteria in both drinking water and surface water sources contaminated by swine CAFOs could contribute to the spread and persistence of both resistant bacteria and antibiotic resistance determinants in humans and the environment.

However, in rural environments, swine CAFOs are not the only potential sources of antibiotic-resistant bacteria. Other sources could include poultry farms, dairy farms, and human sources such as leaking septic tanks and land-applied biosolids. In the present study, an unexpected finding was that up-gradient groundwater samples that were not impacted by the swine CAFO contained significantly higher percentages of erythromycin-resistant *Enterococcus* spp. compared with down-gradient groundwater samples (Table 6). The levels of erythromycin resistance (MICs) in these isolates were not as high as those observed in *Enterococcus* spp. recovered from down-gradient groundwater samples and manure pit samples (Table 4); however, lower-level erythromycin-resistant *Enterococcus* spp. were still present in significant numbers. After sampling was completed, the owners of this up-gradient well informed us that they had experienced problems with their septic tank and field in the past, and perhaps this may have contributed to the presence of erythromycin-resistant *Enterococcus* spp. in their well. However, the role of possible contamination from their septic tank was not confirmed.

Similarly, we found a slightly higher percentage of clindamycin-resistant non–*E. faecalis* in up-gradient surface water samples compared with down-gradient surface water samples (Table 6). Although the difference was not statistically significant and the levels of clindamycin resistance observed in these isolates were lower than those of non–*E. faecalis* recovered from down-gradient surface water samples and manure samples (Table 5), the presence of resistant non–*E. faecalis* in up-gradient surface water suggests that additional sources of resistant bacteria may exist in this environment. These sources could include human septage, companion animals, wild animals, and migratory waterfowl such as Canada geese (Middleton and Ambrose 2005; Sayah et al. 2005). These findings point to the challenges of identifying pristine, uncontaminated control sites for field studies of water sources located in rural settings, where a variety of agricultural and other human and animal activities can introduce pollutants into the surrounding environment.

Limitations of this study concern sample size and antibiotic usage data. A larger sample size would have provided more statistical power to detect differences in percentages of antibiotic-resistant bacteria present in up-gradient versus down-gradient water samples. Additional samples also would have allowed for statistical analyses regarding seasonal variations in water quality. Beyond sample size, this study would have been enhanced if we had been able to obtain specific antibiotic usage data from the swine grower. Unfortunately, the grower did not have this information because the feed used in this facility was pre-mixed and delivered to the swine CAFO by the contracted integrator, which had deemed antibiotic usage data proprietary information. Instead, we used general FDA data describing the types of antibiotics approved for use in U.S. swine production (FDA 2004) to determine which antibiotics to test in this study. In future studies, we plan to improve the sample design (including sample size) so that statistical analyses can be used to explore spatial and temporal variation in antibiotic-resistant bacteria as it relates to surrounding swine CAFOs. However, the difficulties in obtaining specific antibiotic usage data from swine growers could continue to be a challenge for environmental health researchers in the absence of federal and/or state regulations that require growers or integrators to report these data.

## Conclusions

We observed high levels of erythromycin, tetracycline, and clindamycin resistance in *Enterococcus* spp. recovered from surface water and groundwater situated down gradient from a swine CAFO compared with surface water and groundwater located up gradient of the facility. Significantly elevated concentrations of all three fecal indicators tested in this study were also observed in down-gradient surface water and groundwater samples compared with up-gradient surface water and groundwater samples. Although the specific source or sources of these contaminants was not definitively determined, it is likely that swine manure pit leakage or runoff from swine manure–applied fields (Thurston-Enriquez et al. 2005) contributed to these findings. Swine manure management practices, as well as swine feeding practices such as the administration of nontherapeutic levels of antibiotics in swine feeds, continue to pose both environmental and public health challenges, particularly in the immediate environment of swine CAFOs, where vast amounts of swine manure are produced and applied to agricultural fields.

## Figures and Tables

**Table 1:** Sampling dates, sampling locations, and number of samples collected.

				DG SW			
Sampling date	UG GW	DG GW	UG SW	Site 1	Site 2	Site 3	Manure pit
29 Sep 2002					1		
31 Mar 2003	1			1	1	1	
11 Jun 2003	1	1	1	1	1	1	
24 Jun 2003	1	1	1	1	1	1	
30 Jul 2003	1	1	1		1	1	
6 Jan 2004	1	1	1	1	1	1	1

Abbreviations: DG, down gradient; GW, groundwater; SW, surface water; UG, up gradient.

**Table 2:** Concentrations (CFU/100 mL) of fecal indicators in up-gradient (*n* = 4) and down-gradient (*n* = 15) surface water samples and up-gradient (*n* = 5) and down-gradient (*n* = 4) groundwater samples collected in the proximity of a swine CAFO.

Sample type and bacteria	Up-gradient samples [median (range)]^a^	Down-gradient samples [median (range)]^a^	*p*-Value^b^
Surface water			
*Enterococcus* spp.	35 (1–100)	610 (150–4,700)	0.003
*E. coli*	35 (0–40)	400 (10–3,500)	0.007
Fecal coliforms	15 (0–70)	500 (18–2,400)	0.010
Groundwater			
*Enterococcus* spp.	18 (0–67)	85 (16–140)	0.085
*E. coli*	0 (0)^c^	11.5 (3–40)	0.007
Fecal coliforms	0 (0)^c^	20.5 (3–70)	0.007

a Median and range summaries are reported to match more consistently with the nonparametric statistical tests performed.

b *p*-Values were calculated using the two-sample Wilcoxon rank-sum test.

c No *E. coli* or fecal coliforms were detected in these samples on any sampling trip.

**Table 3:** *Enterococcus* spp. isolated from ground-water, surface water, or manure pits located around or beneath a swine CAFO.

*Enterococcus* spp. source	No. of isolates (%)
Up-gradient groundwater	30 (15)
*E. faecalis*	12 (6)
*E. pallens*	1 (0.5)
Other *Enterococcus* spp.	17 (8.5)
Down-gradient groundwater	26 (13)
*E. faecalis*	21 (10.5)
*E. faecium*	1 (0.5)
*E. gallinarum*	1 (0.5)
*E. raffinosus*	1 (0.5)
*E. sulfureus*	1 (0.5)
Other *Enterococcus* spp.	1 (0.5)
Up-gradient surface water	22 (11)
*E. avium*	1 (0.5)
*E. faecalis*	14 (7)
*E. faecium*	2 (1)
*E. hirae*	1 (0.5)
*E. raffinosus*	1 (0.5)
Other *Enterococcus* spp.	3 (1.5)
Down-gradient surface water	107 (53.5)
*E. casseliflavus*	1 (0.5)
*E. dispar*	1 (0.5)
*E. durans*	5 (2.5)
*E. faecalis*	80 (40)
*E. faecium*	12 (6)
*E. pallens*	1 (0.5)
Other *Enterococcus* spp.	7 (3.5)
Manure pit	15 (7.5)
*E. faecalis*	7 (3.5)
*E. hirae*	5 (2.5)
*E. mundtii*	1 (0.5)
*E. sulfureus*	1 (0.5)
Other *Enterococcus* spp.	1 (0.5)
Total	200 (100)

**Table 4:** MIC data (μg/mL) for erythromycin, tetracycline, and vancomycin among *Enterococcus* spp. isolated from groundwater, surface water, or manure pits.

	Erythromycin^a^			Tetracycline^a^			Vancomycin^a^		
*Enterococcus* spp. source	MIC_50_	MIC_90_	MIC range	MIC_50_	MIC_90_	MIC range	MIC_50_	MIC_90_	MIC range
Up-gradient groundwater (*n* = 30)	16	60.8	1–128	< 1	< 1	< 1–32	0.25	58	0.25–> 64
Down-gradient groundwater (*n* = 26)	2	> 256	< 0.5–> 256	2	64	< 1–> 256	4	8	0.25–8
Up-gradient surface water (*n* = 22)	1	2	< 0.5–4	< 1	108.8	< 1–128	2	8	0.5–8
Down-gradient surface water (*n* = 107)	2	> 256	< 0.5–> 256	2	153.6	< 1–> 256	2	8	0.25–> 64
Manure pit (*n* = 15)	> 256	> 256	< 0.5–> 256	128	179.2	< 1–> 256	0.5	2	0.5–2

MIC_50_, MIC required to inhibit the growth of 50% of organisms.

a CLSI resistance breakpoints are as follows: erythromycin, ≥ 8 μg/mL; tetracycline, ≥ 16 μg/mL; vancomycin, ≥ 32 μg/mL (CLSI 2002).

**Table 5:** MIC data (μg/mL) for clindamycin and virginiamycin among *E. faecalis* and non–*E. faecalis* isolated from groundwater, surface water, or manure pits

	Clindamycin^a^			Virginiamycin^a^		
*Enterococcus* spp. source	MIC_50_	MIC_90_	MIC range	MIC_50_	MIC_90_	MIC range
Up-gradient groundwater						
* E. faecalis* (*n* = 12)	8	16	0.06–16	1	1	0.5–1
Non–*E. faecalis* (*n* = 18)	< 0.03	0.06	< 0.03–0.06	0.13	0.13	0.06–0.13
Down-gradient groundwater						
* E. faecalis* (*n* = 21)	8	28.8	0.5–> 128	1	2	1–4
Non–*E. faecalis* (*n* = 5)	8	> 128	4–> 128	0.5	1	0.5–1
Up-gradient surface water						
* E. faecalis* (*n* = 14)	16	32	8–32	1.5	8	0.5–8
Non–*E. faecalis* (*n* = 8)	16	64	4–64	1	2	0.5–2
Down-gradient surface water						
* E. faecalis* (*n* = 80)	16	32	0.06–> 128	1	8	0.13–32
Non–*E. faecalis* (*n* = 27)	> 128	> 128	< 0.03–> 128	1	5.6	0.25–8
Manure pit						
* E. faecalis* (*n* = 7)	128	> 256	64–> 256	8	16	2–16
Non–*E. faecalis* (*n* = 8)	192	> 256	8–> 256	1	32	0.5–32

MIC_50_, MIC required to inhibit the growth of 50% of organisms.

a CLSI resistance breakpoint for clindamycin and virginiamycin is ≥ 4 μg/mL (CLSI 2002).

**Table 6:** Percentage of antibiotic-resistant *Enterococcus* spp. in up-gradient (*n* = 4) versus down-gradient (*n* = 15) surface water samples and up-gradient (*n* = 5) versus down-gradient (*n* = 4) groundwater samples.

	Percent resistant		
Sample type and antibiotic	Up-gradient samples	Down-gradient samples	*p*-Value
Surface water			
Erythromycin	0	18	0.02
Tetracycline	14	33	0.06
Clindamycin^a^	100	89	0.76
Virginiamycin^a^	0	23	0.17
Vancomycin	0	1	0.83
Groundwater			
Erythromycin	67	20	< 0.001
Tetracycline	3	19	0.07
Clindamycin^a^	0	100	< 0.001
Virginiamycin^a^	0	0	—b
Vancomycin	10	0	0.15

*p*-Values were calculated using one-sided Fisher’s exact tests.

a Analyses for clindamycin and virginiamycin resistance were restricted to non–*E. faecalis* isolates.

b No *p-*value could be calculated due to zero counts of virginiamycin-resistant isolates in both sample types.

## References

[b1-ehp0115-001040] Aarestrup FM, Kruse H, Tast E, Hammerum AM, Jensen LB (2000). Associations between the use of antimicrobial agents for growth promotion and the occurrence of resistance among *Enterococcus faecium* from broilers and pigs in Denmark, Finland, and Norway. Microb Drug Resist.

[b2-ehp0115-001040] Anderson ME, Sobsey MD (2006). Detection and occurrence of antimicrobially resistant *E. coli* in groundwater on or near swine farms in eastern North Carolina. Water Sci Tech.

[b3-ehp0115-001040] APHA (1998). Standard Methods for the Examination of Water and Wastewater.

[b4-ehp0115-001040] Bager F, Madsen M, Christensen J, Aarestrup FM (1997). Avoparcin used as a growth promoter is associated with the occurrence of vancomycin-resistant *Enterococcus faecium* on Danish poultry and pig farms. Prev Vet Med.

[b5-ehp0115-001040] Campagnolo ER, Johnson KR, Karpati A, Rubin CS, Kolpin DW, Meyer MT (2002). Antimicrobial residues in animal waste and water resources proximal to large-scale swine and poultry feeding operations. Sci Total Environ.

[b6-ehp0115-001040] Chapin A, Rule A, Gibson K, Buckley T, Schwab K (2005). Airborne multidrug-resistant bacteria isolated from a concentrated swine feeding operation. Environ Health Perspect.

[b7-ehp0115-001040] Chee-Sanford JC, Aminov RI, Krapac IJ, Garrigues-Jeanjean N, Mackie RI (2001). Occurrence and diversity of tetracycline resistance genes in lagoons and groundwater underlying two swine production facilities. Appl Environ Microbiol.

[b8-ehp0115-001040] CLSI (Clinical and Laboratory Standards Institute) (2002). Performance Standards for Antimicrobial Disk and Dilution Susceptibility Tests for Bacteria Isolated from Animals. Approved Standard.

[b9-ehp0115-001040] FDA (U.S. Food and Drug Administration) (2004). FDA Approved Animal Drug Products.

[b10-ehp0115-001040] Haack BJ, Andrews RE (2000). Isolation of Tn916-like conjugal elements from swine lot effluent. Can J Microbiol.

[b11-ehp0115-001040] Jongbloed AW, Lenis NP (1998). Environmental concerns about animal manure. J Anim Sci.

[b12-ehp0115-001040] Krapac IG, Dey WS, Roy WR, Smyth CA, Storment E, Sargent SL (2002). Impacts of swine manure pits on groundwater quality. Environ Pollution.

[b13-ehp0115-001040] Mellon M, Benbrook C, Benbrook KL (2001). Hogging It:! Estimates of Antimicrobial Abuse in Livestock.

[b14-ehp0115-001040] Middleton JH, Ambrose A (2005). Enumeration and antibiotic resistance patterns of fecal indicator organisms isolated from migratory Canada geese (*Branta canadensis*). J Wildl Dis.

[b15-ehp0115-001040] Murray PR, Baron EJ, Jorgensen JH, Pfaller MA, Yolken RH (2003). Manual of Clinical Microbiology.

[b16-ehp0115-001040] Parveen S, Lukasik J, Scott TM, Tamplin ML, Portier KM, Sheperd S (2006). Geographical variation in antibiotic resistance profiles of *Escherichia coli* isolated from swine, poultry, beef and dairy cattle farm water retention ponds in Florida. J Appl Microbiol.

[b17-ehp0115-001040] Sayah RS, Kaneene JB, Johnson Y, Miller R (2005). Patterns of antimicrobial resistance observed in *Escherichia coli* isolates obtained from domestic- and wild-animal fecal samples, human septage, and surface water. Appl Environ Microbiol.

[b18-ehp0115-001040] Singh KV, Murray BE (2005). Differences in the *Enterococcus faecalis lsa* locus that influence susceptibility to quinupristin-dalfopristin and clindamycin. Antimicrob Agents Chemother.

[b19-ehp0115-001040] Thurston-Enriquez JA, Gilley JE, Eghball B (2005). Microbial quality of runoff following land application of cattle manure and swine slurry. J Water Health.

[b20-ehp0115-001040] USDA (2006a). Farms, Land in Farms, and Livestock Operations: 2005 Summary.

[b21-ehp0115-001040] USDA (2006b). Meat Animals Production, Disposition, and Income: 2005 Summary.

[b22-ehp0115-001040] U.S. EPA2000Improved Enumeration Methods for the Recreational Water Quality Indicators: Enterococci, and *Escherichia coli* EPA/821/R-97/004 Washington, DC U.S. Environmental Protection Agency Available: www.epa.gov/nerlcwww/RecManv.pdf [accessed 13 September 2006]

[b23-ehp0115-001040] U.S. EPA (U.S. Environmental Protection Agency) (2002). Drinking Water Contaminants. List of Drinking Water Contaminants & Their MCLs.

[b24-ehp0115-001040] U.S. EPA (2003). Bacterial Water Quality Standards for Recreational Waters (Freshwater and Marine Waters).

[b25-ehp0115-001040] U.S. Geological Survey (2006). USGS Topographic Maps.

[b26-ehp0115-001040] Wegener HC (2003). Antibiotics in animal feed and their role in resistance development. Curr Opin Microbiol.

